# Early suppression of B cell immune responses by low doses of chloroquine and pyrimethamine: implications for studying immunity in malaria

**DOI:** 10.1007/s00436-019-06335-5

**Published:** 2019-05-08

**Authors:** Hayley Joseph, Emily Eriksson, Louis Schofield

**Affiliations:** 1 Division of Population Health and Immunity, Walter and Eliza Hall Institute of Medical Research, 1G Royal Parade, Parkville, VIC 3052 Australia; 2 Department of Medical Biology, The University of Melbourne, Melbourne, VIC 3052 Australia; 3 Australian Institute of Tropical Health and Medicine, James Cook University, Townsville, QLD 4811 Australia

**Keywords:** Malaria, B cells, Chloroquine/pyrimethamine, Memory B cells

## Abstract

Malaria remains a significant worldwide public health problem. To address biological questions, researchers rely on the experimental murine model. For decades, chloroquine (CQ) and pyrimethamine (Pyr) have been used to clear *Plasmodium* infections in experimental animals using standardised accepted protocols and, because of this, drug-treated controls are rarely included. However, there is limited data available on the modulation of anti-malarial immunity, including generation of memory B cells, when these drugs are administered days after malaria infection. We investigated B cell responses to an important malaria glycolipid, glycosylphosphatidylinositol (GPI), and the hapten nitrophenol (NP), with or without standard CQ and Pyr treatment using the murine model. At day 14, CQ/Pyr treatment significantly suppressed the frequency of NP^+^IgG1^+^ memory B cells in NP-KLH-immunised mice. Furthermore, CQ/Pyr-treated NP-KLH-immunised mice did not have significantly higher cellular counts of NP^+^ B cells, germinal centre B cells, nor NP^+^IgG1^+^ memory B cells than naïve mice (CQ/Pyr treated and untreated). CQ/Pyr-treated GPI-KLH-immunised mice did not have significantly higher cellular counts of GPI^+^ B cells than naïve untreated mice. By day 28, this effect appeared to resolve since all immunised mice, whether treated or untreated, had significantly higher B cell proliferative responses than naïve mice (CQ/Pyr treated and untreated) for the majority of B cell phenotypes. The current study emphasises the potential for drug modulation of antigenic B cell responses when using standardised malaria treatment protocols in the experimental murine model. It is recommended that drug-treated controls are included when using experimental malaria infections to address biological questions.

**Electronic supplementary material:**

The online version of this article (10.1007/s00436-019-06335-5) contains supplementary material, which is available to authorized users.

## Introduction

Immunity to malaria is hard to acquire and easily lost (Jeffery 1966), phenomena that contribute to the global persistence of *Plasmodium*. Inadequate B cell memory recall and *Plasmodium* immunomodulation of host responses are likely involved (reviewed in Frosch and John 2012). When studying *Plasmodium*, well-established malaria mouse infection models are often utilised to investigate pathogenesis, immunomodulation, anaemia, liver-stage infections and blood-stage infections (Helegbe et al. 2018; Lau et al. 2014; Ryg-Cornejo et al. 2016b). To do this, administration of low doses (10 mg/kg each) of chloroquine (CQ) and pyrimethamine (Pyr) are crucial to prevent the progression of disease and effectively clear the infection in these mice (Schofield et al. 2017). Standard parasite clearance protocols include simultaneous i.p. injection of the drugs days after malaria challenge, followed by drinking water spiked with anti-malarials (Schofield et al. 2017). Despite the known immunomodulatory effects of CQ and Pyr (Brauner et al. 2017; Khan et al. 2018; Qin et al. 2014; Rynes 1997), many studies do not include parallel drug-treated control mice. This study was specifically designed to observe if the standard protocols used to clear malaria infection alter B cell responses to foreign antigen.

## Materials and methods

To assess immunomodulatory effects of anti-malarial treatment on B cell activation, mice were immunised with the important malaria glycolipid, glycosylphosphatidylinositol (GPI) (Schofield and Hackett 1993), or the hapten nitrophenol (NP), a commonly used antigen to study various aspects of B cell responses (Inamine et al. 2005; Lalor et al. 1992).

### Mouse immunisations

An equal number of female and male inbred C57BL/6 mice (6–8 weeks of age) were used for all experiments and were evenly distributed in different groups. All procedures complied with the Walter and Eliza Hall Institute Animal Ethics Committee requirements.

Mice were immunised with *Plasmodium* GPI conjugated to the carrier protein keyhole limpet haemocyanin (KLH), or NP also conjugated to KLH. Briefly, synthetic GPI was conjugated to maleimide-activated KLH (ThermoFisher Scientific, USA) using 2-iminothiolane and stored at − 80 °C until use (GPI-KLH). 4-Hydroxy-3-nitrophenyl acetyl-Osu (NP-Osu) (Biosearch Technologies, USA) was conjugated to KLH (Sigma-Aldrich, USA; molar ratio between 13 and 20) according to the manufacturer’s instructions and stored at − 20 °C until use (NP-KLH).

For the immunisations, stock antigen vials were thawed and diluted to 20 μg per 100 μL in Hepes Buffered Eagles Essential Medium (HEM). An equal volume of 10% alum (Sigma-Aldrich, USA) was added to the diluted antigen and the pH was adjusted to 6.5 with 1 M sodium hydroxide (NaOH). The solution was washed four times with PBS and resuspended in PBS to 50% of the original volume. For example, if the antigen was originally diluted to 2 mL in HEM, then only 1 mL of PBS was added for the final resuspension. Twenty micrograms per 100 μL of GPI-KLH or NP-KLH precipitated on 10% alum was injected i.p. per mouse.

B cell activation was assessed in two independent experiments at day 14 (prime) and day 28 (boost). Prime experiments involved CQ/Pyr treating half the group of mice at day 5 following immunisation. Mice were subsequently euthanised at day 14. For boost experiments, mice were first immunised at day 0 and half the group of mice were CQ/Pyr treated as above or left untreated. At day 16, mice were boosted with NP-KLH or GPI-KLH as above. All mice included in this set of experiments were euthanised at day 28. Drug treatment was an i.p. injection of CQ (10 mg/kg) and Pyr (10 mg/kg) followed by 5 days of drinking water spiked with CQ (0.6 mg/mL) and Pyr (0.07 mg/mL). The regime was specifically chosen to mimic standardised protocols of parasite clearance in experimental murine models (Schofield et al. 2017). Naïve mice included as controls were either left untreated or administered the same drug treatment as the immunised groups.

Phenotypic analysis of B cells was performed by flow cytometry and detection of antigen-specific ASCs was performed using ELISPOTs. Naïve mice were included as baseline comparisons.

### Flow cytometry

Single cell suspensions were prepared from spleens as previously described (Lee et al. 2017; Ryg-Cornejo et al. 2016a). The following fluorochrome-conjugated monoclonal antibodies were used to stain B cells: Pacific Blue (PB) anti-CD19 (clone 1D3, Ebioscience, USA), allophycocyanin (APC) anti-IgG1 (clone X56), PECy7 anti-CD95 (clone Jo2), and fluorescein isothiocyanate (FITC) anti-CD38 (clone Ab90). IgD^+^ and/or Gr1^+^ cells were determined by Alexa 680 (A680) anti-IgD (clone 1126C) and A680 anti-Gr1 (clone 8C5). Dead cells were excluded using live/dead fixable yellow dead cell staining kits (Life Technologies, USA). Antigen specificity was determined using GPI-BSA-BIO/Streptavidin-PE or NP-PE. Germinal Centre (GC) B cells were defined as CD19^+^IgD^−^CD95^+^CD38^lo^. Within GC populations, antigen-positive and class-switched B cells were defined as GPI/NP^+^IgG1^+^ (CD19^+^IgD^−^CD95^+^CD38^lo^GPI/NP^+^IgG1^+^). Total activated B cells that were antigen positive and class switched were defined as CD19^+^IgD^−^GPI/NP^+^IgG1^+^. Since the adjuvant alum is a strong inducer of Th2 responses (Baz et al. 2012), only the IgG1 class-switched cells were measured. Within this gated population, memory cells were defined as CD19^+^IgD^−^GPI/NP^+^IgG1^+^CD38^+^. Analysis of activated B cells that were specific for antigen was defined as CD19^+^IgD^−^GPI/NP^+^. Gating strategies and representative flow plots are outlined in Suppl. Fig. 1. Fluorescence Minus One (FMO) controls were included to allow accurate gating. Sample analysis was performed on a FortessaX20 flow cytometer and Cell Quest software packages (BD Biosciences, USA). A minimum of 500,000 events was acquired. Data were analysed using FlowJo Version 9.9.6 software (TreeStar).

### ELISPOTs

In vitro differentiation of antibody-secreting cells (ASCs) was performed using ELISPOT, as previously described (Zotos et al. 2010), and visualised using the BCIP/NBT system (KPL, USA). Coating concentrations for NP-BSA and GPI-BSA were optimised at 10 μg/mL.

### Statistical analysis

Statistical analysis included comparing mean values by Mann-Whitney using Prism version 7 software (GraphPad). Statistical significance comparing more than two groups in an analysis was determined using Kruskal-Wallis followed by Dunn’s multiple comparison test as indicated. Statistical analysis included comparisons with the untreated and treated naïve groups.

## Results and discussion

The malaria murine model is a crucial biological tool for understanding host and pathogen interactions. Furthermore, anti-malarial treatment is warranted to ensure mice do not succumb to *Plasmodium* infection yet there is limited data on the effect of these drugs on mounting effective immune responses administered at this timepoint. We wanted to investigate if administration of these drugs, using the widely accepted standard protocol for clearing *Plasmodium* parasites, had an effect on antigen-specific B cell responses in the murine model.

GPI is an important malaria glycolipid with a role in malaria pathogenesis (Schofield and Hackett 1993) and NP is a commonly used antigen to study various aspects of B cell responses (Inamine et al. 2005; Lalor et al. 1992). To measure the B cell priming response and ensuing proliferation following first encounter with foreign GPI-KLH or NP-KLH antigen, mice were euthanised and splenocytes isolated at day 14 post-immunisation. Day 14 was chosen to ensure the detection of a high number of antigen-specific B cells.

**Fig. 1:**
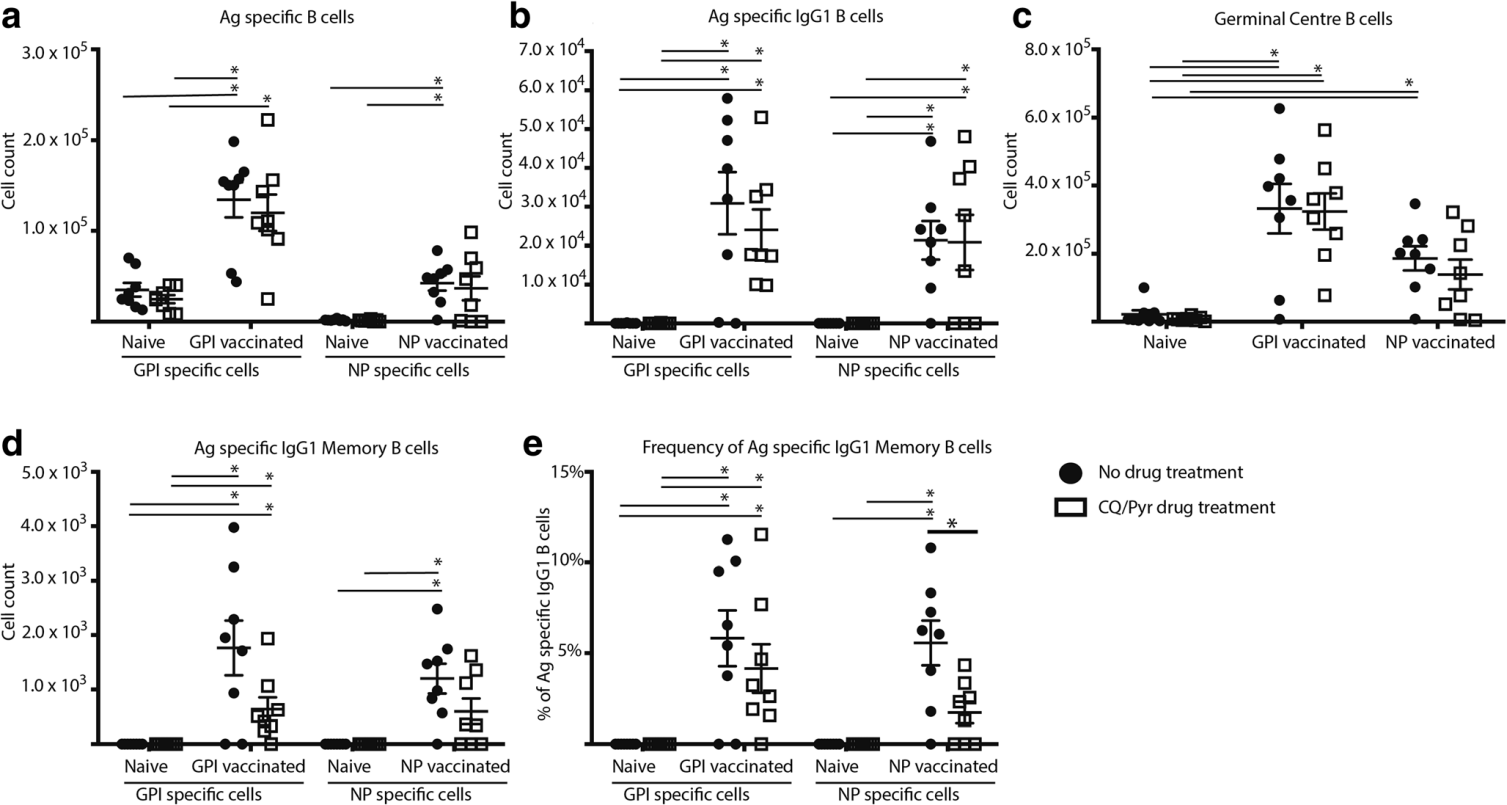
At day 14, CQ/Pyr treatment suppressed NP^+^ MBCs in NP-KLH-immunised mice. Mice were vaccinated at D0 with NP-KLH (*n* = 16) or GPI-KLH (*n* = 16). Naïve mice (*n* = 16) were included as a baseline. At D5, half the mice in each group were treated with CQ/Pyr (open squares). The remaining mice received no treatment (filled circles). Activated B cell populations were analysed at day 14 by flow cytometry and the number of cells per spleen for (A) antigen (Ag)-specific B cells, (B) antigen (Ag)-specific IgG1 B cells, (C) germinal centre (GC) B cells, and (D) antigen (Ag)-specific IgG1 memory B cells was determined. Additionally, the frequency (%) of antigen (Ag)-specific IgG1 MBCs as a proportion of all antigen-specific IgG1 B cells was assessed (E). Figure is a representative of 2 independent experiments. Statistical analysis was performed using the non-parametric Kruskal-Wallis test and significance determined using Dunn’s multiple comparison. Comparing CQ/Pyr treatment vs. no CQ/Pyr was analysed using the non-parametric unpaired Mann-Whitney test. Data are represented as mean ± SEM. *P < 0.05

Immunisation with either NP-KLH or GPI-KLH in the absence of CQ/Pyr treatment induced significant B cell proliferation by day 14 as expected (Fig. 1). Mean B cell counts per spleen in these immunised untreated groups were significantly higher than those in naïve mice (CQ/Pyr treated and untreated) for GPI^+^/NP^+^ B cell counts, GPI^+^/NP^+^IgG1^+^ B cell counts, GC B cell counts, and GPI^+^/NP^+^IgG1^+^ memory B cell (MBC) counts (Fig. 1a–d). Although overall GC cells were readily detectable, the low numbers of GC cells in naïve mice (CQ/Pyr treated and untreated) presented in Fig. 1c represent only activated GC cells as inactivated IgD^−^ B cells were removed from the analysis by gating (Supp. Fig. 1). CQ/Pyr treatment induced a significant suppression of frequency of NP^+^IgG1^+^ MBCs in NP-KLH-immunised mice compared with untreated NP-KLH-immunised mice (Fig. 1e). Although at this timepoint there were no other significant differences between treatment and no treatment, CQ/Pyr appeared to have a subtle effect as few B cell parameters were no longer significantly different to naïve mice (Fig. 1a–d). For the GPI-KLH-immunised drug-treated mice, although the mean GPI^+^ B cell count was significantly higher than that for naïve drug-treated mice, it was not significantly higher than that for naïve untreated mice (Fig. 1a). For the NP-KLH-immunised drug-treated mice, this subtle suppressive effect was observed for NP^+^-specific B cells (Fig. 1a), GC counts (Fig. 1c), and MBCs (Fig. 1d and e). Here, NP-KLH-immunised treated mice did not have significantly higher mean cell counts nor MBC frequencies than naïve mice (CQ/Pyr treated and untreated). This pattern observed for GPI-KLH- and NP-KLH-immunised drug-treated mice was also extended to frequencies of GPI^+^/NP^+^ or GPI^+^/NP^+^IgG1^+^ B cells (Supp. Fig. 2a and b).

**Fig. 2:**
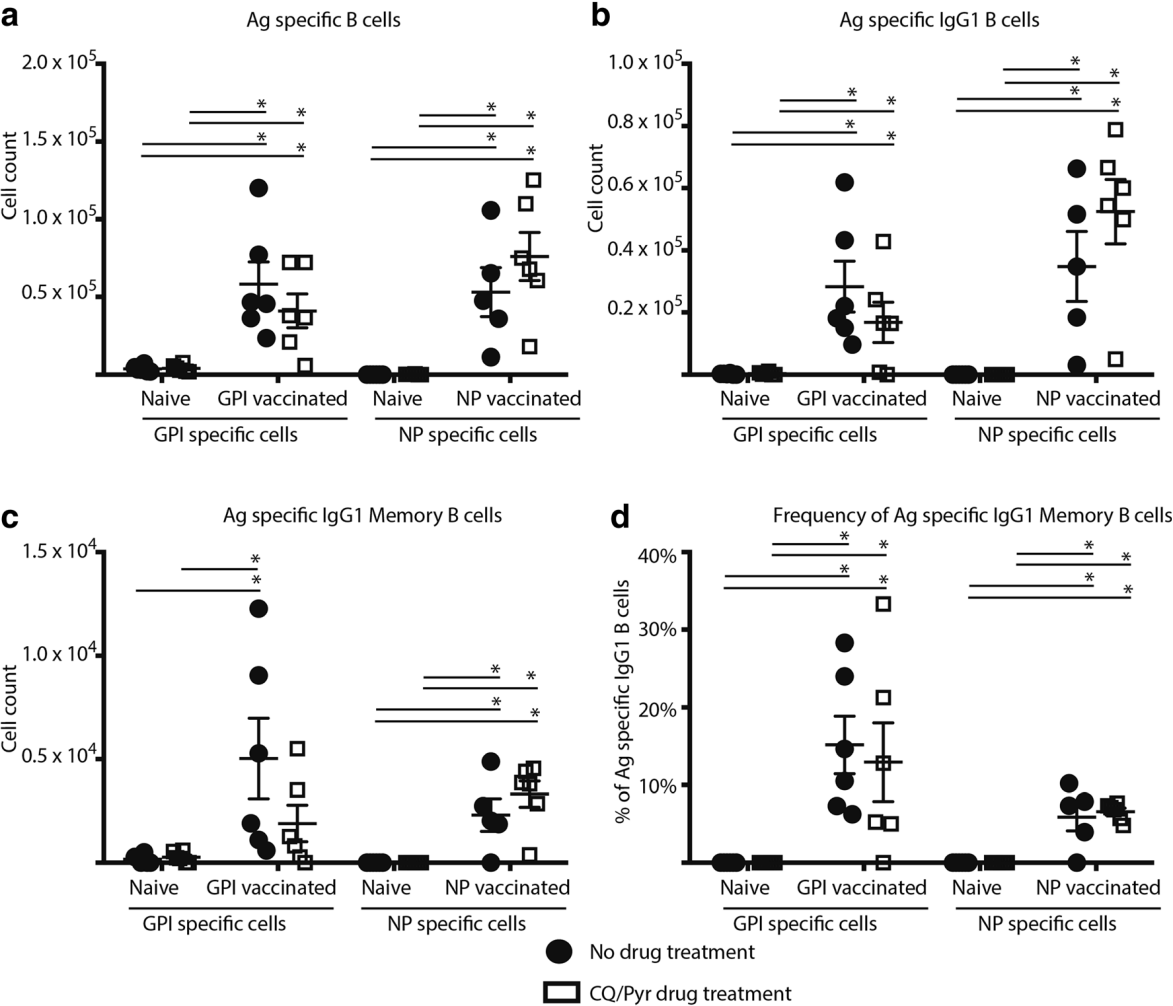
B cell suppression resolves in CQ/Pyr-treated mice. Mice were vaccinated at D0 with NP-KLH (*n* = 18) or GPI-KLH (*n* = 17). Naïve mice (*n* = 18) were included as a baseline. At D5, mice were either treated with CQ/Pyr (open squares) or left untreated (filled circles). At day 16, NP-KLH and GPI-KLH mice were boosted with 20 μg. Mice were euthanised at day 28 and activated B cell populations were analysed by flow cytometry and the number of cells per spleen for (A) antigen-specific B cells, (B) antigen-specific IgG1 B cells, and (C) antigen-specific IgG1 MBCs was determined at day 28. Additionally, the frequency (%) of antigen-specific IgG1 MBCs as a proportion of all antigen-specific IgG1 B cells was calculated (E). All graphs are representative of 2 independent experiments. Statistical analysis was performed using the non-parametric Kruskal-Wallis test and significance determined using Dunn’s multiple comparison. Comparing CQ/Pyr treatment vs. no CQ/Pyr was analysed using the non-parametric unpaired Mann-Whitney test. Data are represented as mean ± SEM. *P < 0.05

Based on these findings, we wanted to further investigate if the significant suppression of the frequency of NP-specific MBCs resolved and if the CQ/Pyr effect was reversible upon a secondary boost with antigen. By day 28, suppression of B cell proliferation in CQ/Pyr-treated NP-KLH-immunised mice resolved, with B cell counts now significantly higher compared with naïve mice (CQ/Pyr treated and untreated) for most B cell subtypes (Fig. 2a–d) and no significant differences in MBC frequencies between CQ/Pyr-treated and untreated NP-KLH-immunised mice (Fig. 2d). The resolution of suppression was also observed for the frequencies of antigen specific and antigen-specific IgG1 B cells for both GPI-KLH- and NP-KLH-immunised mice (see Supp. Fig. 3). Similarly, there were no differences between CQ/Pyr-treated and untreated GPI-KLH-immunised mice for all B cell parameters (Fig. 2a–d). CQ/Pyr-treated GPI-KLH-immunised mice had lower cellular counts of MBCs that were not significantly different to naïve mice (CQ/Pyr treated and untreated). However, the proportion of MBCs of total GPI-specific IgG1 B cells was significantly higher than the proportion of MBCs in naïve mice (CQ/Pyr treated and untreated) (Fig. 2d). Whether this slight low count of GPI^+^ MBCs in CQ/Pyr-treated GPI-KLH-immunised mice increased at a later timepoint was not assessed in the current study.

**Fig. 3:**
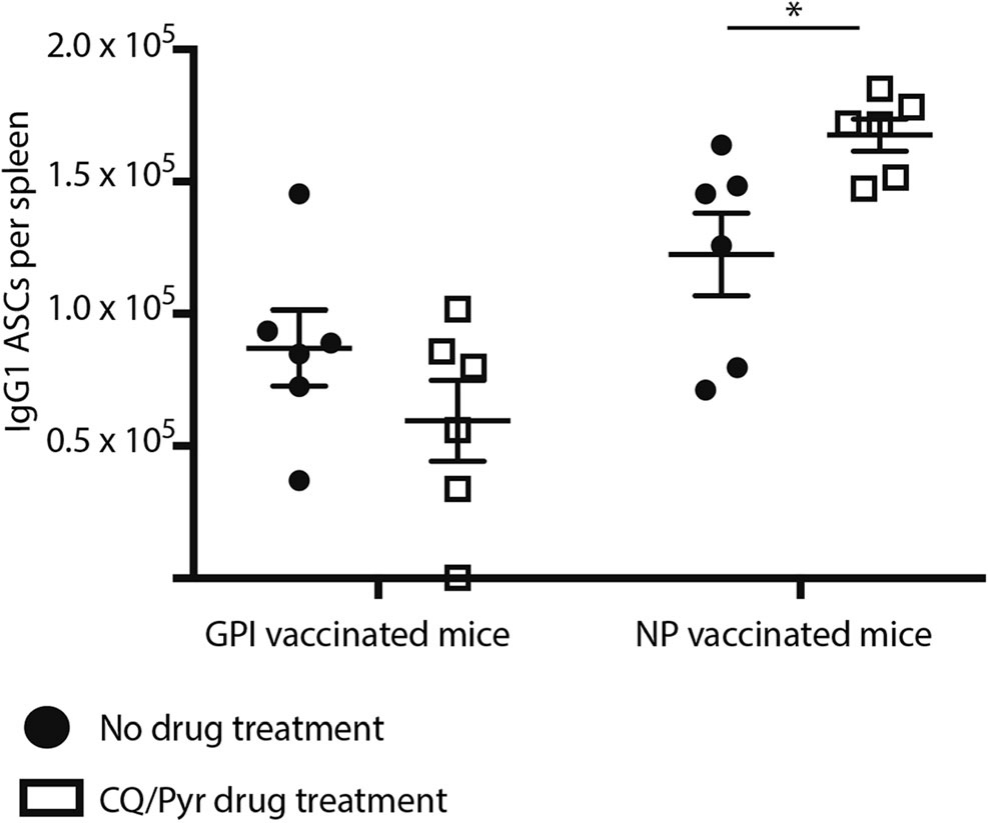
CQ/Pyr did not suppress numbers of antigen-specific IgG1 antibody-secreting cells (ASCs). Mice were vaccinated at D0 with NP-KLH (*n* = 18) or GPI-KLH (*n* = 17). Naïve mice (*n* = 18) were included as a baseline. At D5, mice were either treated with CQ/Pyr (open squares) or left untreated (filled circles). At day 16, NP-KLH and GPI-KLH mice were boosted with 20 μg. Mice were euthanised at day 28. Splenocytes were plated onto pre-coated ELISPOTs to measure numbers of ASCs. Graph is representative of combined data obtained from 2 independent experiments. Comparing CQ/Pyr treatment vs. no CQ/Pyr was analysed using the non-parametric unpaired Mann-Whitney test. Data are represented as mean ± SEM. *P < 0.05

Finally, we wanted to establish if CQ/Pyr treatment impaired the production of functional antigen-specific antibody-secreting cells (ASCs) from the spleen at day 28. CQ/Pyr did not affect the production of anti-GPI IgG1^+^ ASCs nor anti-NP IgG1^+^ ASCs (Fig. 3). Interestingly, there was a significant difference between CQ/Pyr-treated and untreated NP-KLH-immunised mice as a significantly lower count of anti-NP IgG1^+^ ASCs was observed in untreated mice (Fig. 3). As this study chose only one late timepoint, it was not ascertained if the peak of ASCs produced in the spleen occurred earlier in these untreated mice, which could explain the lower count observed.

Our findings are highly suggestive of CQ/Pyr modulating antigen-specific B cell responses when administered according to standard protocols, that is, at day 5 post infection to avoid fatalities due to high parasitaemia (Schofield et al. 2017). This modulation is reflected at day 14 with a significant suppression of the frequency of NP-specific MBCs. Furthermore, although not significant, CQ/Pyr-treated NP- or GPI-immunised mice had lower B cell counts that affected their statistical difference to the naïve populations (CQ/Pyr treated and untreated). By day 28, the observed early significant suppression of NP^+^ MBCs was resolved, as were most of the other B cell counts, suggestive of a transient effect of CQ/Pyr. The current study cannot conclusively ascertain why the effect of CQ/Pyr treatment differed between NP- and GPI-immunised mice. Both NP and GPI were conjugated to KLH to elicit protein T–dependent responses (Swaminathan et al. 2014) and received the same concentration of antigen administered in alum. It is unlikely to be due to potential differences in CQ/Pyr suppression of KLH T cell priming, as T cell priming has been shown to initiate within 8–20 h post vaccination and peak around days 5 to 7 (Mempel et al. 2004), thus occurring prior to drug treatment. Therefore, differences in immunogenicity observed may be due to antigenic size (NP-KLH is far larger than GPI-KLH) or, conceivably, differences in conjugation efficiencies. Further studies will be required to properly address the underlying mechanism behind the observed differences.

Resolution of cellular suppression by CQ/Pyr is consistent with other studies that have shown that the anti-malarial drugs delay humoral responses to chikungunya virus (Roques et al. 2018) and that withdrawal of CQ reverses suppressed humoral responses in rheumatoid arthritis patients (Brauner et al. 2017). Consequently, this research also raises crucial questions concerning not only mounting effective immunity against *Plasmodium* sp. whilst taking malaria prophylaxis but also the efficacy of co-administered vaccines including the malaria vaccine RTS,S. This avenue of research requires further investigation, as does exploring if T cell responses are suppressed by CQ/Pyr treatment using this same model.

Although our study was not designed to ascertain the exact modulation of kinetic effects of CQ/Pyr on B cells, nor the direct effect of antigen activation with simultaneous immunisation and drug treatment, our observations clearly showed that the standard CQ/Pyr treatment protocol of parasite clearance in mice effects B cells. This suggests that drug-treated controls are worthwhile to include in any immune-related measurement when using this drug treatment regimen to account for the effect of CQ/Pyr on immune cells.

## Electronic supplementary material

Supplementary Fig. 1 Representative FACS plot of gating strategies. Gating strategies for measuring activated B cells. Briefly, single cells were gated, followed by exclusion of dead cells. Lymphocytes were gated followed by B cells (CD19^+^). After gating on the single, live, CD19^+^ lymphocytes, the activated CD19^+^ cells were gated on by exclusion of naïve IgD^+^ B cells (dump channel). (PNG 379 kb)

Supplementary Fig. 2 At day 14, CQ/Pyr treatment affected frequencies of antigen specific and antigen specific class-switched NP^+^ B cells. NP-KLH immunised drug treated mice did not have significantly higher frequencies of NP^+^ or NP^+^IgG1^+^ B cells than naïve mice (CQ/Pyr treated and untreated). Graph is representative of combined data obtained from 2 independent experiments. Statistical analysis was performed using the non-parametric Kruskal-Wallis test and significance determined using Dunn’s multiple comparison. Comparing CQ/Pyr treatment vs. no CQ/Pyr was analysed using the non-parametric unpaired Mann-Whitney test. Data are represented as mean ± SEM. *P < 0.05. (PNG 82 kb)

Supplementary Fig. 3 B cell suppression resolves in CQ/Pyr treated mice. Frequencies of antigen-specific and antigen-specific IgG1 B cells were significantly higher in immunised mice (CQ/Pyr treated and untreated) when compared to naïve mice (CQ/Pyr treated and untreated). Graph is representative of combined data obtained from 2 independent experiments. Statistical analysis was performed using the non-parametric Kruskal-Wallis test and significance determined using Dunn’s multiple comparison. Comparing CQ/Pyr treatment vs. no CQ/Pyr was analysed using the non-parametric unpaired Mann-Whitney test. Data are represented as mean ± SEM. *P < 0.05. (PNG 78 kb)
