# Supplementary figures and images for: Early suppression of B cell immune responses by low doses of chloroquine and pyrimethamine: implications for studying immunity in malaria
Source: Parasitol Res. 2019 May 8;118(6):1987–92. doi: 10.1007/s00436-019-06335-5 (PMC6520326; doi:10.1007/s00436-019-06335-5)

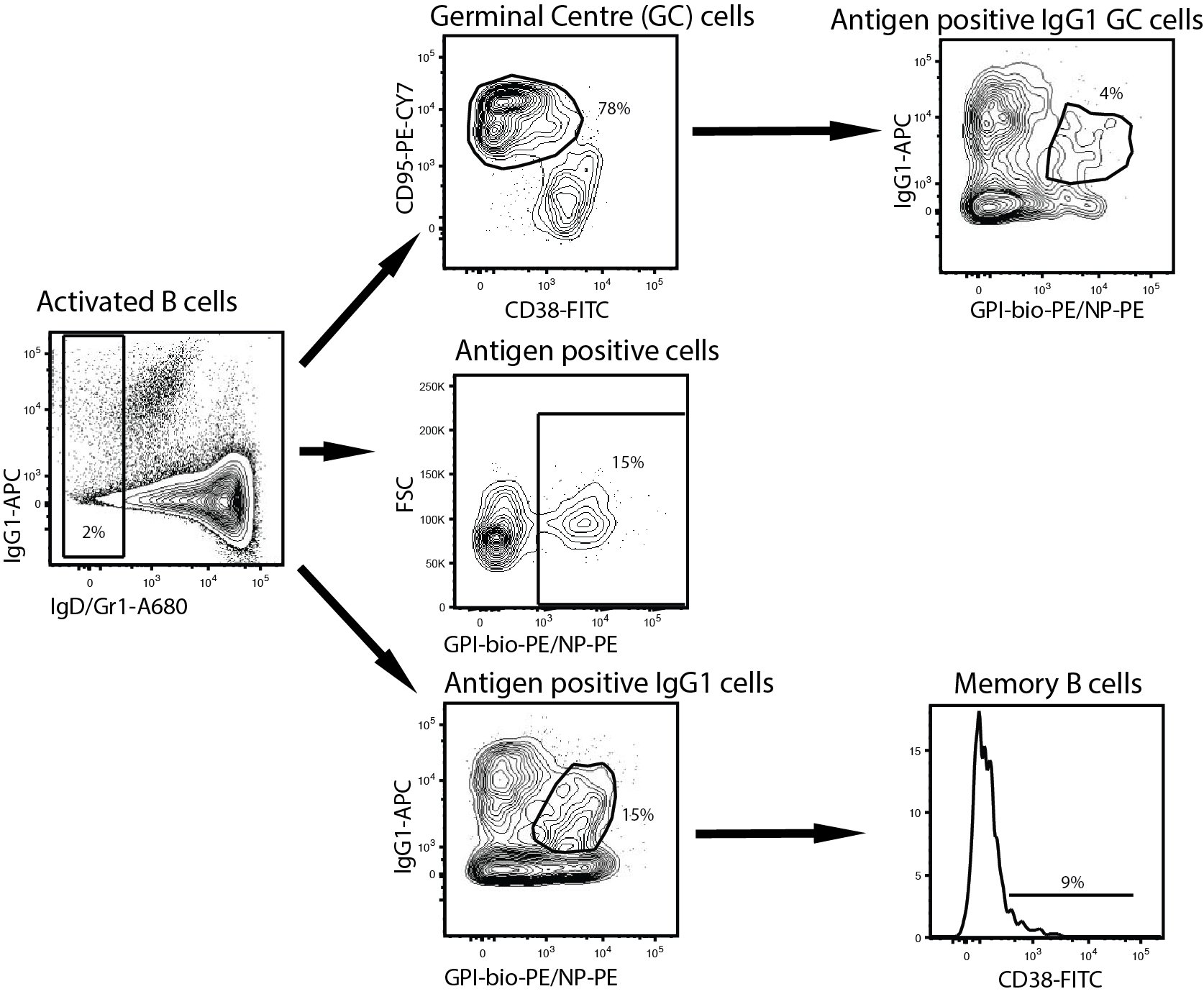

Supplement: Supplementary file 1 — Representative FACS plot of gating strategies. Gating strategies for measuring activated B cells. Briefly, single cells were gated, followed by exclusion of dead cells. Lymphocytes were gated followed by B cells (CD19+). After gating on the single, live, CD19+ lymphocytes, the activated CD19+ cells were gated on by exclusion of naïve IgD+ B cells (dump channel). (PNG 379 kb) [file 436_2019_6335_MOESM1_ESM.png]

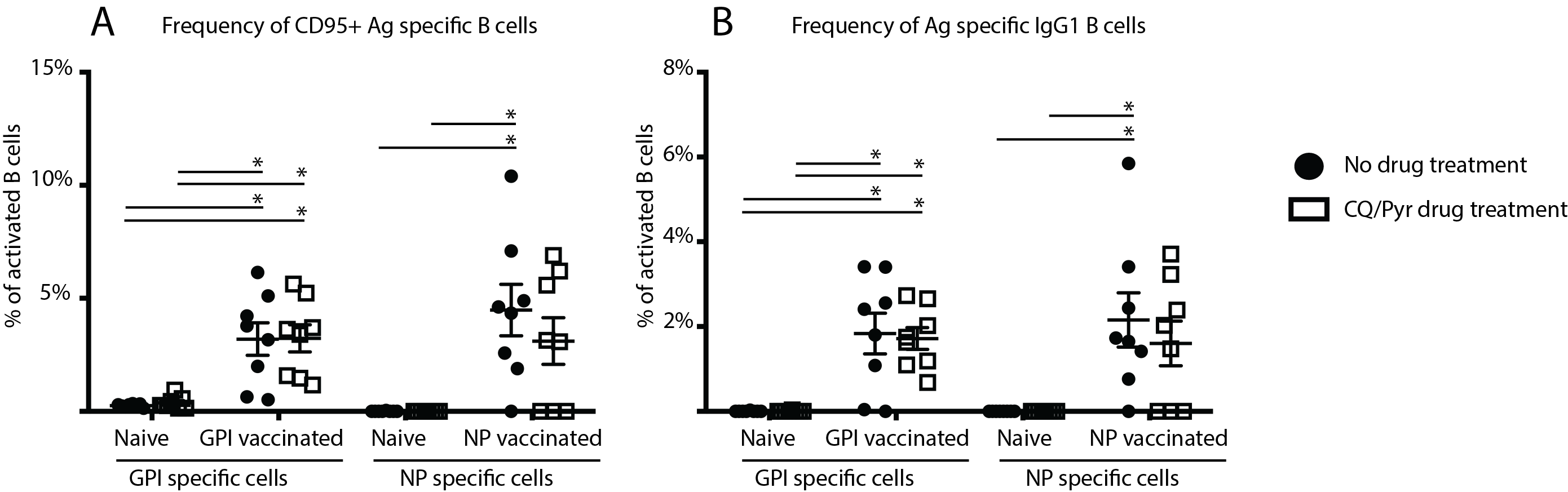

Supplement: Supplementary file 2 — At day 14, CQ/Pyr treatment affected frequencies of antigen specific and antigen specific class-switched NP+ B cells. NP-KLH immunised drug treated mice did not have significantly higher frequencies of NP+ or NP+IgG1+ B cells than naïve mice (CQ/Pyr treated and untreated). Graph is representative of combined data obtained from 2 independent experiments. Statistical analysis was performed using the non-parametric Kruskal-Wallis test and significance determined using Dunn’s multiple comparison. Comparing CQ/Pyr treatment vs. no CQ/Pyr was analysed using the non-parametric unpaired Mann-Whitney test. Data are represented as mean ± SEM. *P < 0.05. (PNG 82 kb) [file 436_2019_6335_MOESM2_ESM.png]

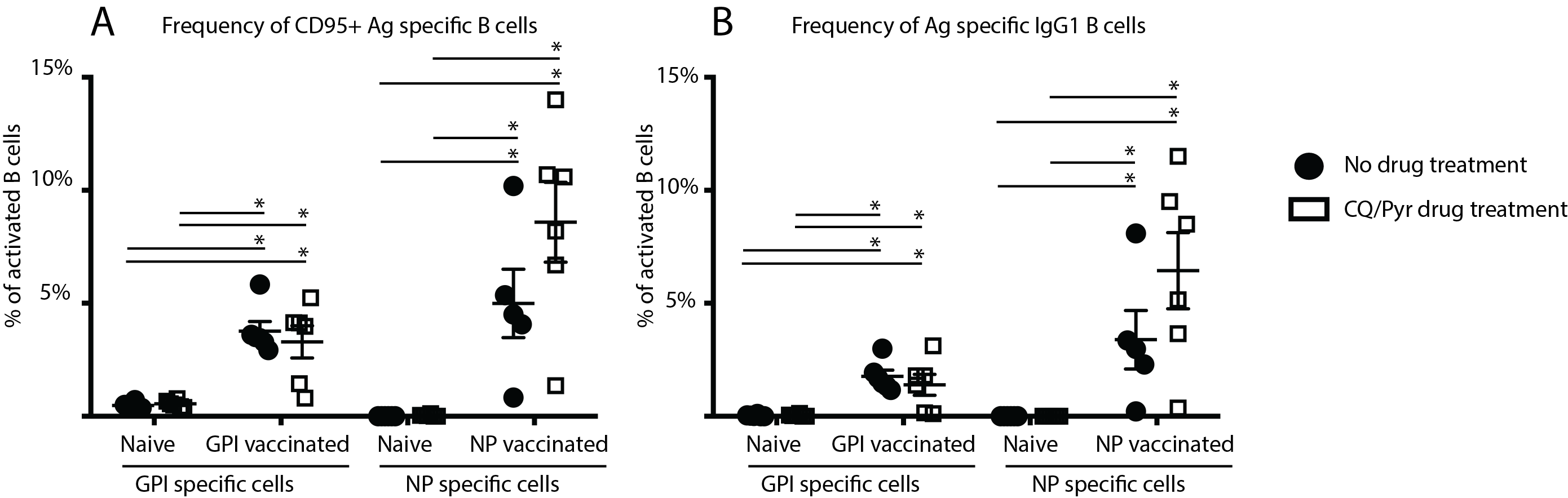

Supplement: Supplementary file 3 — B cell suppression resolves in CQ/Pyr treated mice. Frequencies of antigen-specific and antigen-specific IgG1 B cells were significantly higher in immunised mice (CQ/Pyr treated and untreated) when compared to naïve mice (CQ/Pyr treated and untreated). Graph is representative of combined data obtained from 2 independent experiments. Statistical analysis was performed using the non-parametric Kruskal-Wallis test and significance determined using Dunn’s multiple comparison. Comparing CQ/Pyr treatment vs. no CQ/Pyr was analysed using the non-parametric unpaired Mann-Whitney test. Data are represented as mean ± SEM. *P < 0.05. (PNG 78 kb) [file 436_2019_6335_MOESM3_ESM.png]
